# P-387. Evaluation of Treatment Satisfaction and Experiences Among People With HIV When Switching to Bictegravir/Emtricitabine/Tenofovir Alafenamide From Cabotegravir + Rilpivirine: Results From the Phase 4 EMPOWER Study

**DOI:** 10.1093/ofid/ofaf695.604

**Published:** 2026-01-11

**Authors:** Samir K Gupta, Thomas C S Martin, Cyril Gaultier, Alexandra Kissling, Kathleen Beusterien, Megan Chen, Megan Dunbar, Hui Liu, Brenda Ng, Moti Ramgopal

**Affiliations:** Indiana University School of Medicine, Indianapolis, IN; University of California San Diego, La Jolla, California; BIOS Clinical Research, Palm Springs, California; Oracle Life Sciences, Austin, Texas; Oracle Life Sciences, Austin, Texas; Gilead Sciences, Inc., Foster City, California; Gilead Sciences, Forest City, CA; Gilead Sciences, Inc., Foster City, CA, USA, Foster City, California; Gilead Sciences, Inc., Foster City, California; Midway Immunology and Research Center, Fort Pierce, Florida

## Abstract

**Background:**

Bictegravir/emtricitabine/tenofovir alafenamide (B/F/TAF) is a guideline-recommended treatment for HIV that has shown high levels of efficacy. EMPOWER assessed experiences with and reasons for switching to daily oral B/F/TAF from bimonthly injectable cabotegravir + rilpivirine (CAB + RPV) among people with HIV (PWH).

**Figure:**
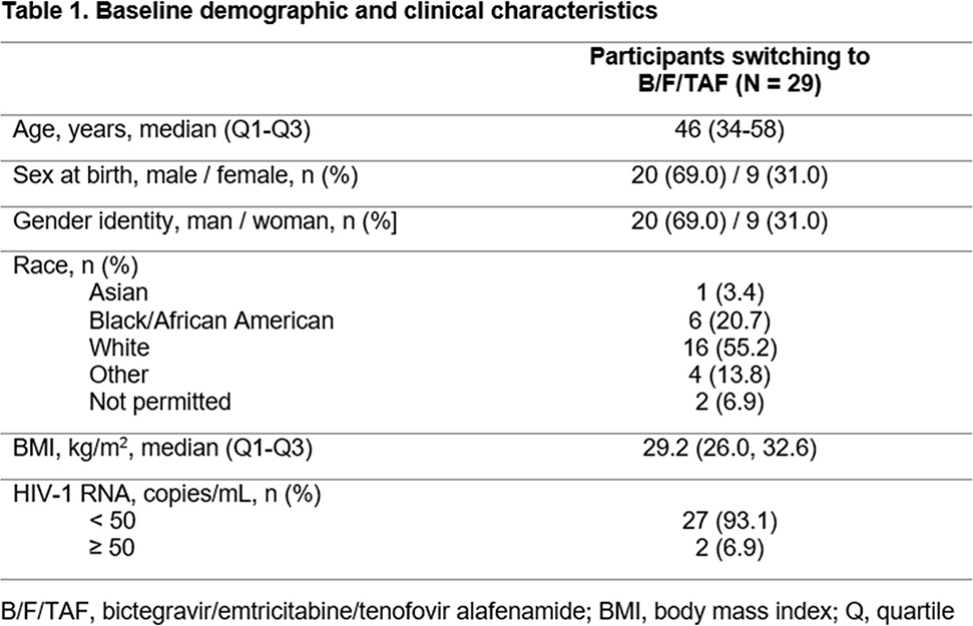

**Figure:**
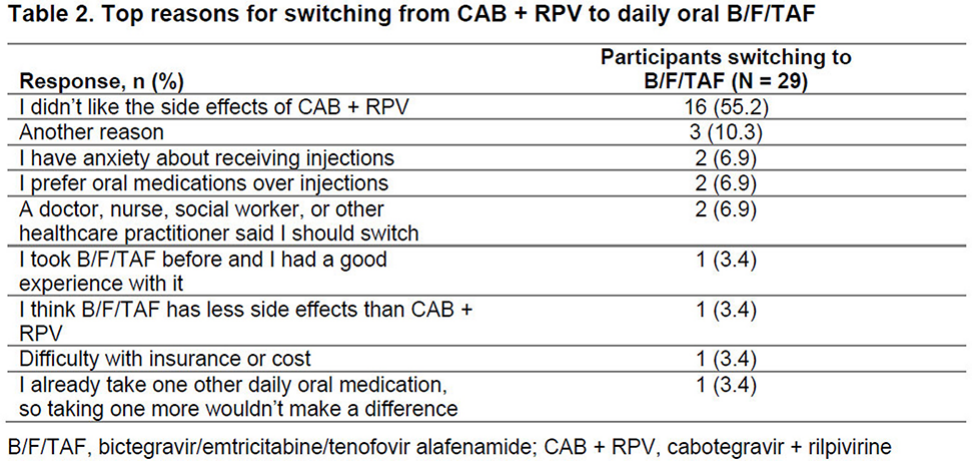

**Methods:**

This Phase 4, single-arm, open-label multicenter study (NCT06104306) assessed switching to B/F/TAF in virologically suppressed PWH unable to continue on CAB + RPV or who expressed a preference to switch to oral therapy. Participants were aged ≥ 18 years, had received ≥ 1 (and no missed) doses of CAB + RPV, and had documented HIV-1 RNA < 50 copies/mL for ≥ 6 months prior to screening. Treatment satisfaction was assessed using the HIV Treatment Satisfaction Questionnaire: status version (HIVTSQs) at baseline (BL), Week (W)12, and W24. Satisfaction with treatment change was assessed using the HIVTSQ: change version (HIVTSQc) at W4. A study-specific questionnaire on reasons for decision to switch therapies was completed at BL and W4. W12 interim analyses were completed using descriptive statistics.

**Figure:**
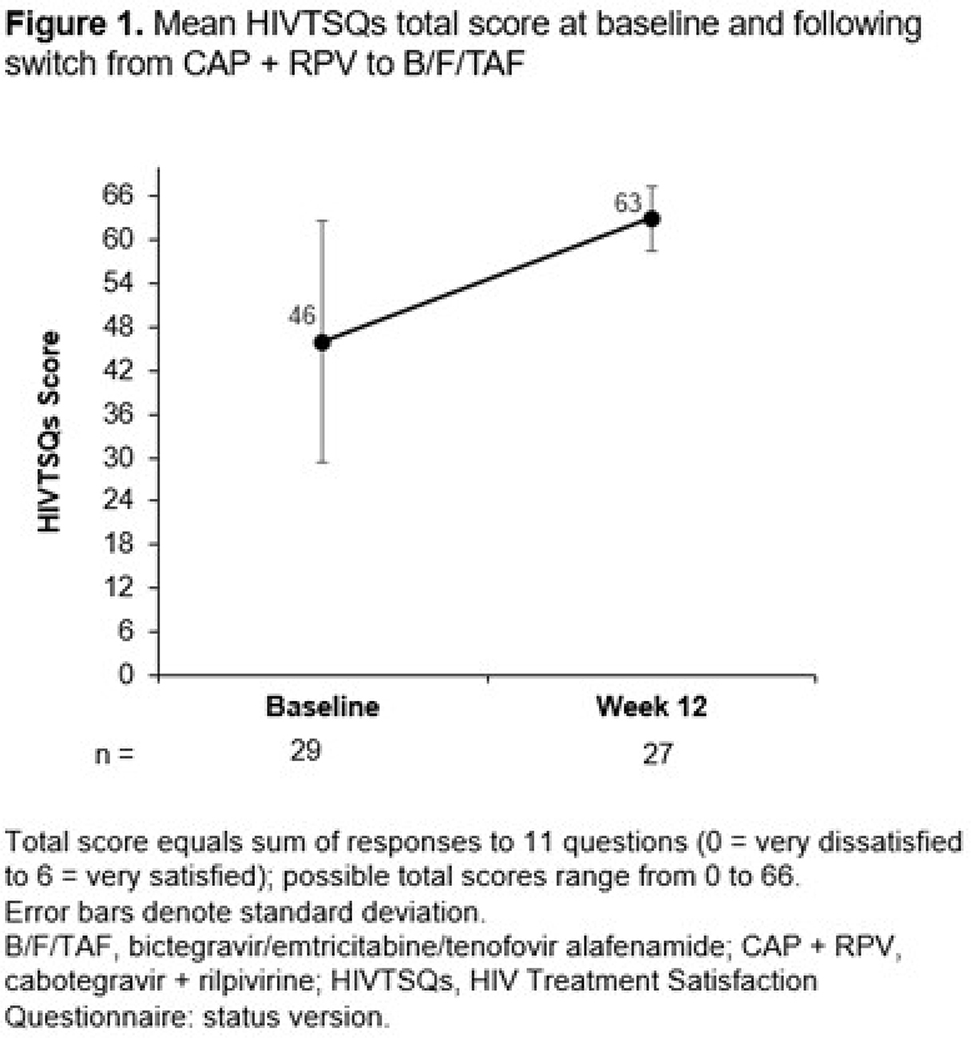

**Figure:**
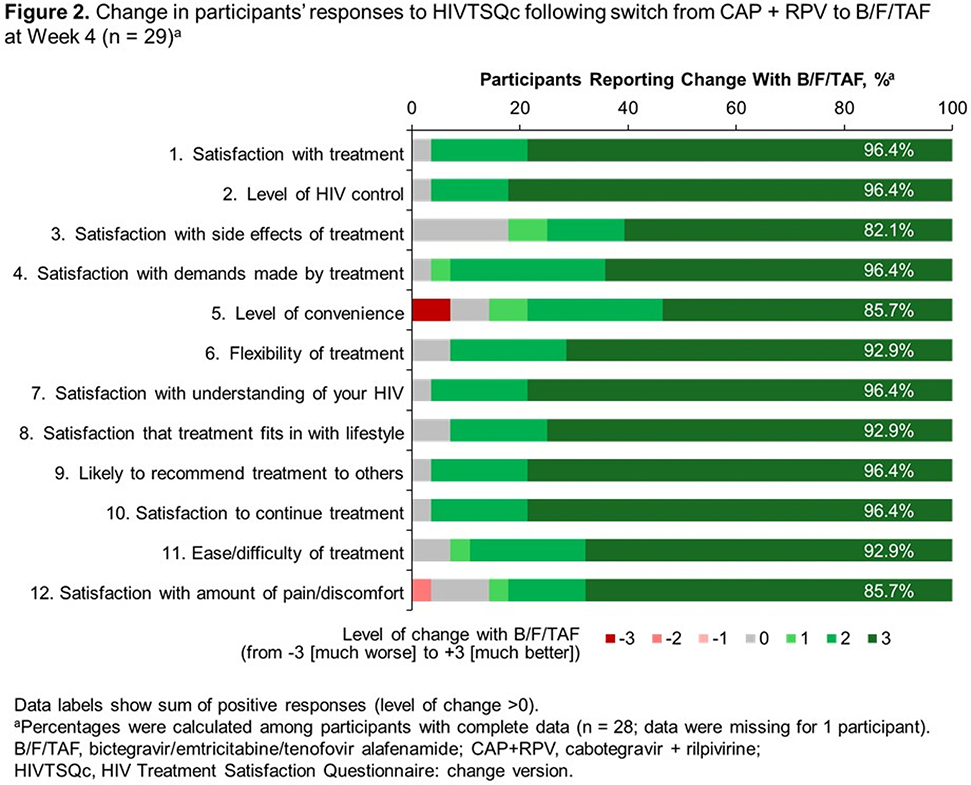

**Results:**

29 participants from North America were included (Table 1). Of those with available data, 21 (84.0%) had ≥ 95% adherence up to W12. All participants achieved HIV-1 RNA < 50 copies/mL at W4 and W12 (missing = excluded). After switching to B/F/TAF, improvements from BL in treatment satisfaction (HIVTSQs) were reported at W12 (Figure 1). At W4, participants (n = 28) reported a mean (SD) increase in treatment satisfaction (HIVTSQc) of 28 (6.8) points . Improvements were seen in all aspects of treatment satisfaction (Figure 2).The most common reason for switching from CAB + RPV was due to side effects (Table 2): 16/28 (57.1%) reported that side effects of CAB + RPV affected their ability to do daily activities. All participants reported feeling hopeful or very hopeful about successfully treating their HIV with B/F/TAF. No grade 3/4 treatment-related adverse events were reported with B/F/TAF.

**Conclusion:**

Participants choosing to discontinue CAB + RPV and start B/F/TAF reported a meaningful increase in treatment satisfaction and fewer side effects. B/F/TAF is an option for PWH wanting to switch from CAB + RPV to a daily oral regimen.

**Disclosures:**

Samir K. Gupta, MD, Gilead Sciences, Inc.: Advisor/Consultant|Gilead Sciences, Inc.: Honoraria|ViiV Healthcare: Advisor/Consultant|ViiV Healthcare: Grant/Research Support|ViiV Healthcare: Honoraria Thomas CS Martin, MD, Gilead Sciences, Inc.: Grant/Research Support Cyril Gaultier, MD, Gilead Sciences, Inc.: Advisor/Consultant|GSK: Advisor/Consultant|Merck: Advisor/Consultant|ViiV: Advisor/Consultant Alexandra Kissling, PhD, Gilead Sciences, Inc.: Advisor/Consultant Kathleen Beusterien, MPH, Oracle America, Inc.: Employee Megan Chen, MSPH, Gilead Sciences, Inc.: Stocks/Bonds (Public Company) Megan Dunbar, PhD, Gilead Sciences, Inc.: Employee|Gilead Sciences, Inc.: Stocks/Bonds (Public Company) Hui Liu, PhD, Gilead Sciences, Inc.: Employee|Gilead Sciences, Inc.: Stocks/Bonds (Private Company) Brenda Ng, PhD, Gilead Sciences, Inc.: Employee|Gilead Sciences, Inc.: Stocks/Bonds (Public Company) Moti Ramgopal, MD, AbbVie: Honoraria|Gilead Sciences, Inc.: Advisor/Consultant|Gilead Sciences, Inc.: Honoraria|Shionogi: Advisor/Consultant|ViiV: Advisor/Consultant|ViiV: Honoraria

